# The role of CD4^+^FoxP3^+^ regulatory T cells in the immunopathogenesis of COVID-19: implications for treatment

**DOI:** 10.7150/ijbs.59534

**Published:** 2021-04-10

**Authors:** Yifei Wang, Jingbin Zheng, Md Sahidul Islam, Yang Yang, Yuanjia Hu, Xin Chen

**Affiliations:** State Key Laboratory of Quality Research in Chinese Medicine, Institute of Chinese Medical Sciences, University of Macau, Macau SAR 999078, China

**Keywords:** COVID-19, SARS-CoV-2, CD4^+^FoxP3^+^ regulatory T cells, immunopathology

## Abstract

The severe cases of Coronavirus Disease 2019 (COVID-19) frequently exhibit excessive inflammatory responses, acute respiratory distress syndrome (ARDS), coagulopathy, and organ damage. The most striking immunopathology of advanced COVID-19 is cytokine release syndrome or “cytokine storm” that is attributable to the deficiencies in immune regulatory mechanisms. CD4^+^FoxP3^+^ regulatory T cells (Tregs) are central regulators of immune responses and play an indispensable role in the maintenance of immune homeostasis. Tregs are likely involved in the attenuation of antiviral defense at the early stage of infection and ameliorating inflammation-induced organ injury at the late stage of COVID-19. In this article, we review and summarize the current understanding of the change of Tregs in patients infected with severe acute respiratory syndrome coronavirus 2 (SARS-CoV-2) and discuss the potential role of Tregs in the immunopathology of COVID-19. The emerging concept of Treg-targeted therapies, including both adoptive Treg transfer and low dose of IL-2 treatment, is introduced. Furthermore, the potential Treg-boosting effect of therapeutic agents used in the treatment of COVID-19, including dexamethasone, vitamin D, tocilizumab and sarilumab, chloroquine, hydroxychloroquine, azithromycin, adalimumab and tetrandrine, is discussed. The problems in the current study of Treg cells in COVID-19 and future perspectives are also addressed.

## Introduction

The infection of severe acute respiratory syndrome coronavirus 2 (SARS-CoV-2) is initiated by the binding of the virus to angiotensin-converting enzyme 2 (ACE2) and the internalization of the complex to the host cells 1, 2. Patients with Coronavirus disease 2019 (COVID-19) manifest a range of symptoms of varying severity from no symptoms (asymptomatic) to severe pneumonia, which can progress to acute respiratory distress syndrome (ARDS), metabolic acidosis, septic shock, coagulopathy, organ failure, and even to death 3, 4. Deaths of COVID-19 patients are frequently associated with a cytokine storm syndrome 5, a common feature of several infectious and non-infectious disease, including H5N1 influenza, severe acute respiratory syndrome coronavirus (SARS-CoV), Middle-East respiratory syndrome coronavirus (MERS-CoV), Epstein-Barr virus, cytomegalovirus, group A streptococcus, and graft-versus-host disease 6, 7. Marked elevation of cytokine levels such as interleukin (IL)-6, IL-7, IL-1, IL-10, IL-2, granulocyte colony-stimulating factor, C-X-C motif chemokine ligand 10, monocyte chemoattractant protein-1, macrophage inflammatory proteins-1 alpha, and tumor necrosis factor (TNF) in patients with severe COVID-19 is the characteristics of cytokine storm 8. Moreover, clinical data indicate that lymphopenia (i.e., decrease in CD4^+^ and CD8^+^ T cells, NK cells, and B cells) is common in COVID-19 patients as well, which may be associated with the negative prognosis of COVID-19 patients 4, 9, 10. Remarkably, dysregulation of the T cell populations is strongly correlated with severity in COVID-19 patients 11, 12.

CD4^+^ FoxP3^+^ Tregs are a subset of potent immunosuppressive cells that play a vital role in maintaining immune homeostasis and in the prevention of autoimmune responses 13, 14. Presumably, Tregs are required to induce immune tolerance specific to SARS-CoV-2 and suppress the excessive inflammation in patients, while they also likely attenuate the host defense capacity to eliminate infection of SARS-CoV-2. The possibly important role of Tregs in immunopathology and Tregs may represent therapeutic targets have spurred the investigators to examine the change of Tregs in COVID-19 patients, especially those with ARDS 11, 15, 16.

In this article, we review and summarize the current understanding of the change of Tregs in patients infected with SARS-CoV-2 and discuss the potential role of Tregs in the immunopathology of COVID-19. The emerging concept of Treg-targeting therapies, including both adoptive Treg transfer and a low dose of IL-2 treatment, is introduced. Furthermore, the potential Treg-boosting effect of therapeutic agents used in the treatment of COVID-19 is analyzed. We also discuss the problems in the current study of Treg cells in COVID-19 and future perspectives.

## Overview of immunopathology in COVID-19 infection

Previously, there were two major coronavirus (CoVs) outbreaks, e.g., SARS-CoV and MERS-CoV, which resulted in fatal respiratory diseases with alarming morbidity and mortality 17. The current worldwide outbreak of coronavirus disease 2019 (COVID-19) is caused by a new lineage beta CoVs, namely SARS-CoV-2 3. The genome of SARS-CoV-2 contains 3′ and 5′ untranslated regions and open reading frames that code the non-structural and structural proteins essential for the virus, including spike, nucleocapsid, membrane, and envelope structural proteins 18. A genome analysis from a patient with COVID-19 showed nearly 90% nucleotide identity with SL-CoVZXC21, the bat SARS-related-CoV and 82% identity with human SARS-CoV Tor2 and SARS- CoV BJ01 2003 19. However, the external subdomain of SARS-CoV-2 Spike's receptor binding domain just shares less than half of amino acid identity with other SARS-related coronaviruses 19. The infection of SARS-CoV-2 was due to the binding of S protein to ACE2 expressed by the host cells, which caused a further release of the RNA of SARS-CoV-2 1, 20.

Innate immunity is the first line of defense against viruses. RNA of SARS-CoV-2 that works as a pathogen‐associated molecular pattern can be recognized by host-pathogen recognition receptors, including cytosolic RIG-I-like receptors and extracellular and endosomal Toll-like receptors (TLR) 21. Then specific signal adapter proteins like NF-κB and interferon-regulatory factor family protein are activated and finally trigger the production of type I interferons, which is considered the most important cytokine for antiviral defense 1, 21. In the alveoli of the lungs of COVID-19 patients, the infiltration of CD68^+^ macrophage is increased, accompanied by the production of cytokines (IL-6, TNFα, and IL-10) 22. More than half of ARDS in all COVID-19 patients manifested macrophage activation syndrome with high levels of TNF and IL-6 in the circulation 23. In the peripheral blood of COVID-19 patients, the proportion of CD14^+^CD16^+^ inflammatory monocytes in CD45^+^ leukocytes is increased, especially in the patients with severe-pulmonary-syndrome 24. These inflammatory monocytes have the capacity to produce high levels of GM-CSF and IL-6 24. As shown by a study on 452 patients, markedly elevated levels of IL-6 and slightly enhanced levels of TNF and IL-2R, while no marked change of IL-1β, were observed in COVID-19 patients 11. Notably, lymphopenia is a common clinical manifestation in patients with COVID-19 3. In addition to inflammatory macrophages, the number of neutrophils is also increased in the circulation, which can be an indicator to predict the severity of COVID-19 8. The neutrophil-to-lymphocyte ratio is a clinical inflammation biomarker. This ratio is elevated in the peripheral blood of patients with COVID-19, and the enhanced ratio is associated with the severity of infection at the early stage of disease 25. Patients treated in the ICU tend to have higher cytokines, including TNF, IL-2, IL-7, IL-10, G-CSF, and monocyte chemoattractant protein-1 in the plasma, and the elevation of these cytokines is related to the progress of disease 3. These cytokines further activate immune cells and increase the pulmonary infiltration that causes lung damage or even ARDS.

CD4 and CD8 T cells are important for the elimination of pathogens through cell-mediated immune responses. Although the absolute number of CD4 and CD8 T cells is lower in patients with COVID-19, these two subsets of T cells are hyperactivated 26. A high rate of asymptomatic infected individuals who could not be detected by serological testing may be due to the inhibition of virus replication through immune cell-mediated responses 27. A recent study shows that in mild COVID-19, SARS-CoV-2 induces strong responses of CD8 T cells, characterized by the expression of granzyme A, B, and perforin, without obvious responses of CD4 T cells 28. However, the responses of cytotoxic CD8 T cells are not detectable in COVID-19 patients over the age of 80 years, which may explain why the incidence of severe or critically severe illness was high in elderly patients 28. It was shown that SARS-CoV-2-specific CD8 T cells express IFNγ and CD107a, a degranulation marker of CD8 T cells, while SARS-CoV-2-specific CD4 T cells had the capacity to produce IFNγ, IL-2, and TNF 29. A recent study found that CD4 T cells in patients with COVID-19 express higher levels of CD38, HLA-DR, and Ki-67 30. With a memory and Th1 phenotype, CD4 T cells in COVID-19 could express IFNγ, the signature cytokine of Th1 responses 30. These CD4 T cells could respond to the second peptide pool PepMix 2 of SARS-CoV-2, which spans the entire S protein and contains putative MHC-II epitopes in SARS-CoV. However, although early T cell responses against SARS-CoV-2 are likely to be protective, SARS-CoV-2 manages to develop a mechanism to escape T cell-mediated immunity 31, 32, presumably partially through the activation or expansion of Tregs. Therefore, Treg cells may play dual and/or biphasic roles: downregulation of T cell-mediated immune responses against SARS-CoV-2 in the early stage of infection as well as dampening the excessive inflammation in severe COVID-19 patients in the late stage.

## The role of Tregs in the immunopathology of COVID-19: current understanding

CD4^+^FoxP3^+^ regulatory T cells (Tregs) are a subset of CD4^+^ T cells that typically develop in the thymus (aka. thymus-derived Treg cells or naturally-occurring Treg cells) and can also be induced in the periphery (aka.* In vitro*-induced Treg cells) 33. Tregs play a crucial role in the maintenance of immune homeostasis and inhibition of autoimmune inflammatory responses by potently inhibiting the activation, proliferation, and effector functions of other immune cells 34. The identification of human Tregs based on surface markers remains challenging. Earlier studies found that human Treg cells express high levels of CD25 (IL-2 receptor alpha chain), e.g., CD25^+/hi^, and are negative or low expression of CD127 (IL-7 receptor alpha chain), e.g., CD127^-/lo^ 35, 36. Consequently, the conjunction of CD25^+/hi^ and CD127^-/lo^, as well as lineage marker CD4, are frequently used as surrogate surface makers of FoxP3-expressing Treg cells in human 37, 38. Our own previous study revealed that the combination of CD25 and TNFR2 is able to identify more of FoxP3-expressing Tregs in human peripheral blood 39. FoxP3 is a master nuclear transcript factor that determines the development, function, and phenotype of Treg cells. To date, FoxP3 remains to be the most reliable and specific marker of Treg lineage 40. Moreover, Tregs also express different surface and intracellular markers including co-inhibitory/co-stimulatory molecules (CTLA-4, PD-1, TIM3, LAG3, TIGIT, ICOS, and CD28), Toll-like receptors (such as TLR1, -2, -4, -5, -6, -7, -8 and -9), chemokine receptors (CCR2, -4, -5, -6, -7 and -8, CXCR3 and -4) and TNF receptor superfamily (TNFR2, OX40, 4-1BB, GITR, and FAS) 41. The mechanism of Treg-mediated immunosuppression has been extensively studied. A number of putative mechanisms have been reported, including the production of immunosuppressive cytokines (IL-10, IL-35, and TGFβ), consumption of IL-2, induction of death of effector cells via granzyme and perforin, inhibition of the activation of antigens presenting cells (APC), metabolic disruption (such as the generation of adenosine) and others 42. However, the universal molecular mechanism is still elusive, and further research is needed. We (Xin Chen and Joost J. Oppenheim) for the first time found and reported that TNF through TNFR2 signaling could enhance the expression FoxP3, promote the proliferative expansion and increase the immunosuppressive function of Treg cells 43. Interestingly, TNF preferentially upregulates the expression of TNFR2, along with other members of the TNF receptor superfamily (OX40, 4-1BB, and FAS), on the surface of Tregs 44. We also demonstrated that TNFR2 and its major signaling component IKKα are crucial for the *in vivo* function and phenotypical stability of Tregs 45, 46.

It was previously reported that, in respiratory virus infection, Treg cells could quench cytokine storm, ameliorate virus-induced pneumonia and acute lung injury 47-49. In human and mouse acute lung injury, accumulation of Tregs is attributable to the attenuation of immunopathology by inhibition of the innate immune responses 49. Therefore, it is likely that Tregs are protective in COVID-19 patients with excessive inflammation and cytokine storm.

Several studies indicate that the number of immunosuppressive Tregs in peripheral blood is moderately increased in patients with mild COVID-19 16, 50, 51. Elevated levels of Tregs are also observed in some patients with more severe disease 50, 51. For example, it was reported that the proportion of conventional T cells, B cells, and NK cells in COVID-19 patients were reduced, while Tregs (defined by CD4^+^CD25^+^CD127^-^ ) were increased by 7% and 5% in the mild and severe cases, respectively 50. Since their surface expression of CD25 was up-regulated, and CD127 was down-regulated, Tregs in COVID-19 may be activated, with an enhanced suppressive activity 50. Interestingly, soluble CD25 was increased in the peripheral blood of COVID-19 patients, while its ligand IL-2 was also increased 3, 52. In mechanically ventilated COVID-19 patients with ARDS, in sharp contrast to the lymphopenia in both subsets of CD4 and CD8 T cells, the proportion of Treg cells (defined by CD4^+^FoxP3^+^) was increased in the lungs and peripheral blood mononuclear cells (PBMC) 53. The degree of Treg recruitment into the lungs of COVID-19 patients may determine the severity of the disease since patients with more Treg cells experienced milder disease 54, 55. Interestingly, it was reported that in convalescent COVID-19 patients, the expression of FoxP3 in circulating CD4 T cells was higher than that in uninfected individuals 56. Another study demonstrates that, although the total number of Tregs (CD4^+^ CD25^+^ CD127^-^) in an asymptomatic infected person did not change, the percentage of activated Tregs (CD45RA^-^ FoxP3^hi^) was increased 4.4-fold, as compared with healthy controls 57. In the rhesus monkey model infected with SARS-CoV-2, the proportion of CD4^+^FoxP3^+^ Tregs, as well as CD4^+^ IFNγ^+^ Th1 and CD4^+^ IL-4^+^ Th2 cells, in the lungs was increased at 3 days post-infected (dpi). Furthermore, an increased proportion of Tregs in PBMCs could also be observed from 3 to 21 dpi (*P*<0.05, 21 dpi), while the proportions of Th1 and Th2 cells were decreased as the severity of the illness was increased in the following days 55. It was demonstrated by a previous study that, in mouse experimental acute lung injury model, increased accumulation of Treg cells in bronchoalveolar lavage fluid (BALF) mediated the resolution of lung injury by induction of neutrophil apoptosis, macrophage efferocytosis, and decrease of fibrocyte recruitment 58.

Although plenty of studies indicate the proportion or number of Tregs is increased in patients with COVID-19 (especially those with the milder disease), it was also reported that the number of Tregs is reduced in the patients. For example, it has been reported that Tregs (CD3^+^ CD4^+^ CD25^hi^ CD127^lo^ FoxP3^+^) in PBMCs were markedly decreased in severe COVID-19 patients 59-61. The result of single-cell analysis showed that FoxP3 expression was remarkedly reduced in severe patients with COVID-19, although the expression of CD25 was higher 62. High expression of CD25 on T cell correlates to furin protease that eases the entry of virus particles 62. A recent study examined Tregs (CD4^+^ FoxP3^+^ CD25^+^) in PBMC derived from the COVID-19 patients treated in ICU and found a marked decline in the number of Tregs with a decreased expression of FoxP3 mRNA and immunosuppressive cytokines (IL-10 and TGFβ) 63. Another study reported that the expression of CD25 and FoxP3 mRNA by Tregs was markedly decreased in COVID-19 patients, compared to healthy donors 60. Interestingly, a report shows that the frequency of Tregs (CD3^+^ CD4^+^ CD25^hi^ CD127^lo^) was significantly declined in critically ill patients, while that was increased in mild and severe cases after SARS-CoV-2 infection 51. Other studies also found that the proportion of Tregs in CD4 T was reduced in adults and children with severe COVID-19 11, 64, 65. These studies conclude that a reduced number of Tregs, as well as increased Th17 responses, may be attributable to the excessive inflammation and pathogenesis of COVID-19. Moreover, a recent study reported that the proportion of Tregs (defined by CD3^+^ CD4^+^ CD25^+^ FoxP3^+^) and the levels of FoxP3 expression by Tregs were increased in COVID-19 patients, and that was correlated with poor outcome 66. In addition to expressing immunosuppressive molecules, these Tregs also expressed pro-inflammatory cytokine IL-32, suggesting that Treg cells in acute patients with severe disease may inhibit anti-viral T cell responses while promoting inflammatory responses 66.

**Figure 1:**
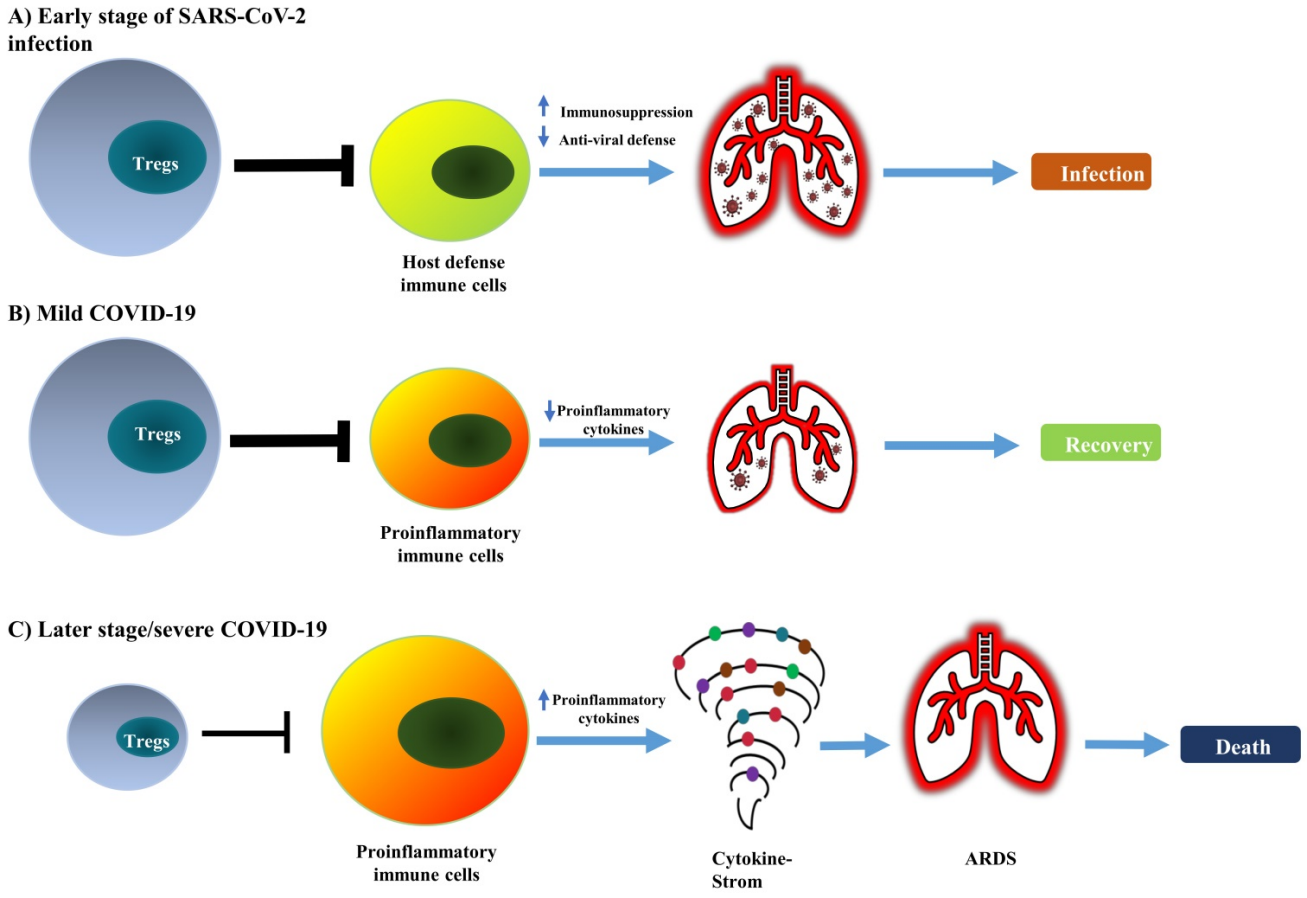
** Conceptual model of dual and biphasic roles of Tregs in SARS-CoV-2 infection and immunopathology of COVID-19.** In SARS-CoV-2 infection, Tregs are likely to play detrimental and beneficial dual roles and biphasic roles in the early and late stages of the disease. **(A)** The augmented Treg population in the early stage of infection potently suppresses the mobilization of host defensive immune cells such as Th1 cells and CD8^+^ CTLs, consequently reducing anti-viral immune responses. **(B)** An increased number of Tregs could attenuate the inflammatory responses and quench the cytokine storm; this can promote the recovery of patients. **(C)** In severe COVID-19, depletion of Tregs can enhance the activation of pro-inflammatory immune cells and production of pro-inflammatory cytokines that cause cytokine storm and lead to lung injury, ARDS, and eventually the death of patients.

It is worth noting that some studies did not observe any change in the frequency of Tregs (CD4^+^ CD25^+^ FoxP3^+^) in the peripheral blood of COVID-19 patients 67, 68, including in cancer patients infected with SARS-CoV-2 69. Therefore, current reports on the change of absolute and relative number of Treg cells in COVID-19 patients remains controversial (as summarized in Table 1). This should be mainly caused by the different criteria used in the identification of Tregs and the observation was made in different stages of the disease. Nevertheless, the majority of studies indicate that Treg cells in COVID-19 patients are activated, which presumably represents a negative feedback mechanism of the immune system to avoid damage of self-tissues by activated immune cells. It is also possible that, in the early stage of infection, an increased number of activated Tregs may reduce antiviral defense by potently inhibiting the immune responses against SARS-CoV-2. In contrast, a reduction in the number of impaired functions of Tregs in severe cases or later stages of the disease may contribute to the excessive production of pro-inflammatory cytokines that lead to ARDS (schematically shown in Figure 1).

## Treg-targeted treatment for COVID-19: promising results from case reports

The excessive inflammatory responses in COVID-19 patients with ARDS suggest that Tregs should be protective. Therefore, the immunosuppressive property of Tregs may be harnessed to curtail the cytokine storm seen in the critically ill COVID-19 patients 70, 71. In fact, Treg-targeted treatments, including the adoptive transfer of Tregs and rIL-2 administration, have been proposed and studied.

### Adoptive Transfer of Tregs

Adoptive Treg transfer is a promising cellular therapy for the treatment of graft-versus-host-disease and autoimmune diseases 72, 73. Importantly, previous studies show that this treatment is effective in a variety of preclinical models of ARDS 49, 74. Moreover, animal studies indicate that the transfer of Tregs could improve the survival rate of mice infected with coronavirus-induced encephalitis and reduce virus-induced cardiac fibrosis 75, 76. Therefore, infusion of *ex vivo* expanded Treg cells may be able to restore Treg homeostasis in patients with insufficient Treg activity caused by SARS-CoV-2 infection and consequently ameliorate life-threatening manifestations by inhibiting excessive inflammation and quenching cytokine storm.

This idea was tested and supported by a recent case study that examined the effect of adoptive transfer of allogeneic HLA-matched umbilical cord blood-derived Tregs 77. In this report, two critically ill COVID-19 patients with ARDS were intravenously administered with allogeneic Tregs derived from cord blood (1×10^8^ cells per dose). Prior to treatment with Tregs, both patients received tocilizumab (anti- IL-6 receptor antibody), vasopressors, and the first patient also received hydroxychloroquine and broad-spectrum antimicrobial agents. After 2 rounds (Patient 1; on day 13 and 17) to 3 rounds (Patient 2; on day 8, 11, and 15) of Treg infusion, the conditions of both patients markedly improved, accompanied by reduced levels of proinflammatory cytokines (IL-6, TNF, IFNγ, IL-8, IL-12, and MCP-4). None of them demonstrated any infusion reaction, inflammatory rebound, or other adverse reaction 77. Therefore, infusion of cord blood-derived Treg cells (designated as CK0802) may serve as an off-the-shelf cellular therapy in the treatment of COVID-19 with ARDS. The efficacy and safety are under evaluation by an ongoing clinical trial (NCT04468971). Furthermore, RAPA-501-ALLO hybrid TREG/Th2 cells with the potential to reduce inflammation and mediate a protective effect on tissues are under study in another clinical trial as an off-the-shelf therapy for patients with severe COVID-19 and ARDS (NCT04482699) 78.

### Recombinant interleukin-2 (rIL-2)

It has been reported that COVID-19 patients have elevated levels of circulating soluble IL-2 receptors 11, 15, 52, 79. Soluble IL-2 receptors may restrain the expansion of Treg cells in COVID-19 patients by reducing the bioavailability of IL-2 to Treg cells 52. A recent case study reported that the treatment with rIL-2 (1 million IU per day) could markedly increase the number of lymphocytes in the peripheral blood (p<0.01) 80. Furthermore, the level of C-reactive protein was decreased after rIL-2 treatment (although no statistically significant, p>0.05) 80. Prompted by the previous studies showing that *in vivo* treatment with low-dose IL-2 (≤1 million IU per day) was able to specifically induce Treg expansion in patients with type 1 diabetes and graft-versus-host disease 81, 82, a clinical trial was registered to study the efficacy of low-dose IL-2 in the treatment of ARDS related to COVID-19 (NCT04357444).

Thus, two treatments aiming to boost Treg activity in COVID-19 patients have been studied and reported. Their pros and cons are listed in Table 2. Although the outcomes of such treatments are promising based on the case reports, ongoing large-scale clinic trials may provide more convincing results to verify the efficacy and safety of such therapies.

## Upregulation of Treg activity by COVID-19 therapeutic agents used in clinic

### Dexamethasone

Glucocorticoids have been widely used in the treatment of inflammatory and autoimmune diseases, including asthma, chronic obstructive pulmonary disease (COPD), and rheumatic diseases 83, 84. The common glucocorticoids used in the clinic including prednisone, prednisolone, budesonide, and dexamethasone 83. Previously, glucocorticoids were widely used in the treatment of SARS and MERS, which share some similarities with COVID-19 85. On 2nd September 2020, an interim guideline regarding the use of dexamethasone and other glucocorticoids for COVID-19 patients was declared by World Health Organization, which strongly recommends glucocorticoids including dexamethasone for severe and critical COVID-19 patients 86. Dexamethasone proves effective in the treatment of critically ill COVID-19 patients with hypoxic respiratory failure 87. In a controlled, open-label RECOVERY trial, dexamethasone treatment markedly reduced mortality 85. The immunosuppressive effect of dexamethasone is attributable to its efficacy in the treatment of COVID-19 88.

Our previous study showed that *in vivo* treatment with dexamethasone increased in the proportion of Treg cells and an elevated ratio of Tregs/Teffs 89. We also found that dexamethasone could allow Treg expansion induced by IL-2, and the combination of IL-2 and dexamethasone synergistically increased the number of Tregs and inhibited the development of experimental autoimmune encephalomyelitis (EAE) in mice 90. Dexamethasone was also reported to improve the function of Treg in human patients with Graves' disease 91. Therefore, dexamethasone may be able to boost Treg activity in COVID-19 patients.

### Vitamin D

A number of recent studies suggest that vitamin D deficiency was associated with an increased risk of infection, mortality, and even requirement for intensive care among hospitalized patients of COVID-19 92, 93. For example, a study based on bioinformatics and systems biology found that vitamin D suppressed cytokine storm and enhanced antiviral response by binding with its receptor 94. It could either inhibit the expression of pro-inflammatory cytokines by blocking the TNF-induced NFκB1 signaling pathway or initiated the expression of interferon-stimulating genes for the antiviral defense program through activating the interferon-induced JAK/STAT signaling pathway 94. To date, there are 76 registered clinical trials in ClinicalTrials.gov to evaluate the effect of vitamin D on COVID-19 95, 96.

Although the exact correlation between vitamin D deficiency and susceptibility of SARS-CoV-2 infection remains elusive, the immunomodulatory effect of vitamin D in viral disease and autoimmune disease provides a strong rationale for using it in COVID-19 97, 98. There is evidence that vitamin D has the capacity to inhibit the production of pro-inflammatory cytokines (IL-6 and TNF) and increase the production of immunosuppressive cytokines, and this property of vitamin D could be harnessed to curtail excessive inflammation triggered by viral infections 99, 100. Vitamin D can directly act on cells in innate and adaptive immune systems to regulate various immune pathways 101. In fact, a wide spectrum of immune cells, including macrophages, neutrophils, dendritic cells, B and T lymphocytes, express vitamin D receptor and these cells can convert vitamin D into its active form that can modulate the adaptive and innate immunity 102, 103. It has been reported that Vitamin D induces the generation of tolerogenic dendritic cells that have the capacity to induce IL-10-producing CD4 T cells and antigen-specific Tregs 104.

Additionally, previous studies have demonstrated that dendritic cells induced by vitamin D3 treatment express high levels of transmembrane TNF (mTNF), and such dendritic cells could induce antigen-specific Tregs through the interaction of mTNF-TNFR2 105. In addition, vitamin D3 inhibited T cell-mediated inflammation and promoted the proliferation of Treg cells 106, 107. Vitamin D supplements can also upregulate the expression of FoxP3, IL-10, and TGF-β1 gene in Tregs and increase the number and boost the function of Tregs 108, 109. A recent animal study reported that the treatment with vitamin D resulted in the marked reduction of the methylation of the conserved non-coding sequence 2 region of FoxP3 109. Moreover, there is experimental and clinical evidence that high vitamin D levels are associated with an enhanced ratio of Treg/total T cells 110, 111. Oral administration of vitamin D3 can increase the absolute number of Tregs (CD4^+^CD25^+^FoxP3^+^CD127^lo^) in the peripheral blood and enhance the immunosuppressive activity of Tregs in healthy adults and patients with autoimmune inflammatory diseases 111, 112. Therefore, vitamin D may have a beneficial effect in the management of SARS-CoV-2 infection by boosting Treg activity.

### Tocilizumab and Sarilumab

Tocilizumab and sarilumab are monoclonal antibodies specific to the IL-6 receptor that is used to control cytokine release syndrome, which is characterized by a marked increase in proinflammatory cytokines, including IL-6 113, 114. The effect of tocilizumab on patients with COVID-19 was investigated in China previously. This study found that the treatment was effective in the majority of patients and the responsive patients recovered within 2-weeks 115. Another study also found a similar effect 116. The therapeutic effect of sarilumab, another antibody specific for IL-6R, on COVID-19 is under study in a clinical trial in the US (NCT04315298).

There is some evidence that blockade of IL-6 receptor can promote Treg activity. For example, it was shown that in murine models of autoimmune diseases, anti-IL-6RAb induces the generation of Treg while inhibiting the differentiation of Th17 and Th1 cells 117. In patients with rheumatoid arthritis, the proportions of CD4^+^CD25^+^CD127^lo^ Tregs and HLA-DR^+^ activated Tregs are markedly increased after tocilizumab therapy 118. Therefore, tocilizumab and sarilumab may also promote the generation of Tregs in COVID-19 patients, and this possibility should be further studied.

### Chloroquine and Hydroxychloroquine

Chloroquine is an effective schistosome insecticide with a quinoline skeleton that can fight against malaria parasites (mainly *Plasmodium falciparum*) 119. Hydroxychloroquine, one of the analogs synthesized on the base of the chloroquine molecule, is also frequently used in malaria treatment 120. Chloroquine was listed in the Chinese "Guideline on diagnosis and treatment of COVID-19 (Trail sixth edition)" in Feb 2020 as a recommended therapeutic agent 121. US Food and Drug Administration also granted an emergency authorization to use chloroquine and hydroxychloroquine for the treatment of COVID-19 122, although its efficacy remains controversial 123, 124. It has been reported that chloroquine treatment promoted the expansion of Treg cells in EAE mice and consequently inhibited the development of EAE 125. In patients with systemic lupus erythematosus (SLE), *in vitro* experiment indicated that chloroquine could rebalance Th17 cells and Treg cells, while *in vivo* study showed that hydroxychloroquine treatment restored the balance of the immune system and increased the levels of FoxP3 in Treg cells 126. Therefore, it is interesting to ask if the therapeutic effect of chloroquine or hydroxychloroquine, if any, is partially based on their activities in promoting Treg activity.

### Azithromycin

Azithromycin (9-deoxo-9a-aza-9a-methyl-9a-homoerythromycin) is a bacteriostatic macrolide with immunoregulatory activity and mTOR inhibitory activity. The result of a clinic trial (EU Clinical Trials Register: 2020-000890-25) in France shows that azithromycin could enhance the efficacy of hydroxychloroquine in the treatment of COVID-19 123. Previously, it was reported that azithromycin, same as rapamycin, could promote Treg phenotype, including FoxP3 expression, in bulk Tregs 127. It was also reported that *in vivo* treatment with azithromycin could result in the expansion of Tregs in mouse model of graft-versus-host disease 128. Thus, the combination of azithromycin and hydroxychloroquine may synergistically increase the number of Tregs in COVID-19.

### Adalimumab

Adalimumab is an anti-TNF monoclonal antibody approved by the US Food and Drug Administration for the treatment of autoimmune diseases including rheumatoid arthritis, Crohn's disease and others 129. A number of case reports showed that adalimumab treatment received by patients with autoimmune diseases has a beneficial effect in the prevention of the clinical progression of severe COVID-19 3, 130-133. This provides a strong rationale for the clinical trial to examine the effect of anti-TNF therapy on COVID-19 134. Two randomized controlled trials (NCT04705844 & ChiCTR2000030089) have been registered to evaluate the efficacy of adalimumab in the treatment of COVID-19 patients with ARDS. Interestingly, it was reported that adalimumab could upregulate the expression of transmembrane TNF on monocytes, and through the TNF-TNFR2 pathway, promoted the activation and expansion of Tregs in rheumatoid arthritis patients 135-138. Therefore, the Treg-boosting effect of adalimumab may contribute to its preventive effect on severe COVID-19 progression.

### Tetrandrine

Tetrandrine is an anti-inflammatory bis-benzylisoquinoline alkaloid isolated from the root of *Stephania tetrandra* S Moore, a traditional Chinese herb 139. This compound was approved for the treatment of silicosis in China 140. It has also been used as an analgesic and diuretic agent 140. Recently, researchers urged to repurpose tetrandrine as adjuvant therapy for the treatment of COVID-19, which might reduce pulmonary fibrosis, and a phase IV clinical trial has been registered (NCT04308317) 141. Tetrandrine is a known blocker of the two‐pore channel, and this property of tetrandrine can inhibit the host cell entrance of ebola virus, MERS-CoV, and SARS-CoV-2 spike protein pseudovirions 142-144. Recently, we reported that inhibition of the two‐pore channel in APC by tetrandrine enhanced the expression of mTNF on APC and consequently induced the proliferative expansion of Tregs via TNFR2 signaling 145, 146. Further, we also found that tetrandrine potently inhibited the differentiation of Th1, Th2 and Th17 cells, while sparing the differentiation of Treg cells 139. Thus, in addition to interfering with cellular entrance and replication of the virus, tetrandrine may attenuate the excessive inflammation in severe COVID-19 by stimulation of Treg proliferative expansion.

Taken together, therapeutic agents currently used in the clinic for the treatment of COVID-19 have the potential to promote Treg activity (summarized in Table 3). It is possible that the Treg-boosting effect of these agents may contribute to their therapeutic effect and this possibility merit future study.

## Conclusions and perspectives

To date, the reports regarding the change of Treg cell number in COVID-19 patients remains controversial (Table 1). It should be mainly caused by the use of different markers in the identification of Tregs since CD25^+/hi^, CD127^-/lo^, FoxP3^+^, and their various combinations were used. Furthermore, Tregs number is likely different in COVID-19 patients in the various stages or with various severity of illness. CD25^+/hi^ and CD127^-/lo^ are just surrogate surface markers of FoxP3-expressing Tregs; thus, it would be ideal only to use CD4 and FoxP3 to identify Tregs in the future study 37, 38. Nevertheless, most studies indicate that Treg cells in COVID-19 patients are activated. Unfortunately, the suppressive function of Treg cells in COVID-19 patients has not to be evaluated with a standard *in vitro* Treg functional assay.

Immunosuppressive Tregs cells and proinflammatory Th17 cells are two major subsets of CD4 T cells. If the balance of Th17/Treg cell shifts towards Th17 cells the release of other pro-inflammatory cytokines will be triggered and inflammatory responses in COVID-19 patients will be exaggerated 147. It was shown that the proportion of Tregs was markedly decreased, while the proportion of CCR6^+^ Th17 cells was increased in COVID-19 patients 148, 149. TGF-β is required for the differentiation of both Tregs and Th17 cells 150. IL-6 can promote the differentiation of Th17 cells through phosphorylating and activating STAT3, while STAT3 can downregulate TGF-β-induced Foxp3 expression and consequently inhibit Tregs cell differentiation 151. Intriguingly, the levels of IL-6 were markedly elevated in COVID-19 patients 11, and that can tip the balance of Treg/Th17 toward Th17 responses.

To harness the potential beneficial and protective effect of immune suppressors on severe cases, Treg cell-targeted treatments have studied, as shown in two case reports 77, 80. Adoptive transfer of cord blood-derived allogenic Tregs had a clear beneficial effect in critically ill patients 77. Hopefully, the efficacy and safety profile of such treatment could be verified by large-scale clinical trial (NCT04468971). The low dose of IL-2 was proposed as a treatment for COVID-19. However, the levels of IL-2 are increased in severe COVID-19 patients 3, 152. Thus, the possibility that rIL-2 may further fuel inflammatory responses should be closely monitored since the activated pro-inflammatory T cells also express CD25 (the alpha chain of IL-2 receptor). It was reported that dexamethasone could permit IL-2-induced expansion of Tregs while potently inhibiting the activation of Teff cells, and dexamethasone is used in the treatment of COVID-19 as an immunosuppressive agent 87, 90. Thus, it would be reasonable to propose the combination of dexamethasone and rIL-2 in the treatment of critically ill COVID-19 patients. Hopefully, this combination therapy can reduce the dose of dexamethasone as well as its potential side effect 90.

Providing enhanced Treg activity is beneficial in severe COVID-19; other agents with the capacity to promote the induction or expansion of Treg cells should also be considered. For example, rapamycin and all-trans retinoic acid can promote TGFβ-induced differentiation of Tregs from naïve CD4 cells 153, 154. TNFR2-agonist antibody 155, 156 and IL-2/anti-IL-2 monoclonal antibodies 157-159 can stimulate the expansion of pre-existing naturally occurring Tregs. These agents may also have beneficial effect in the treatment of COVID-19 by boosting Treg activity.

**Figure 2:**
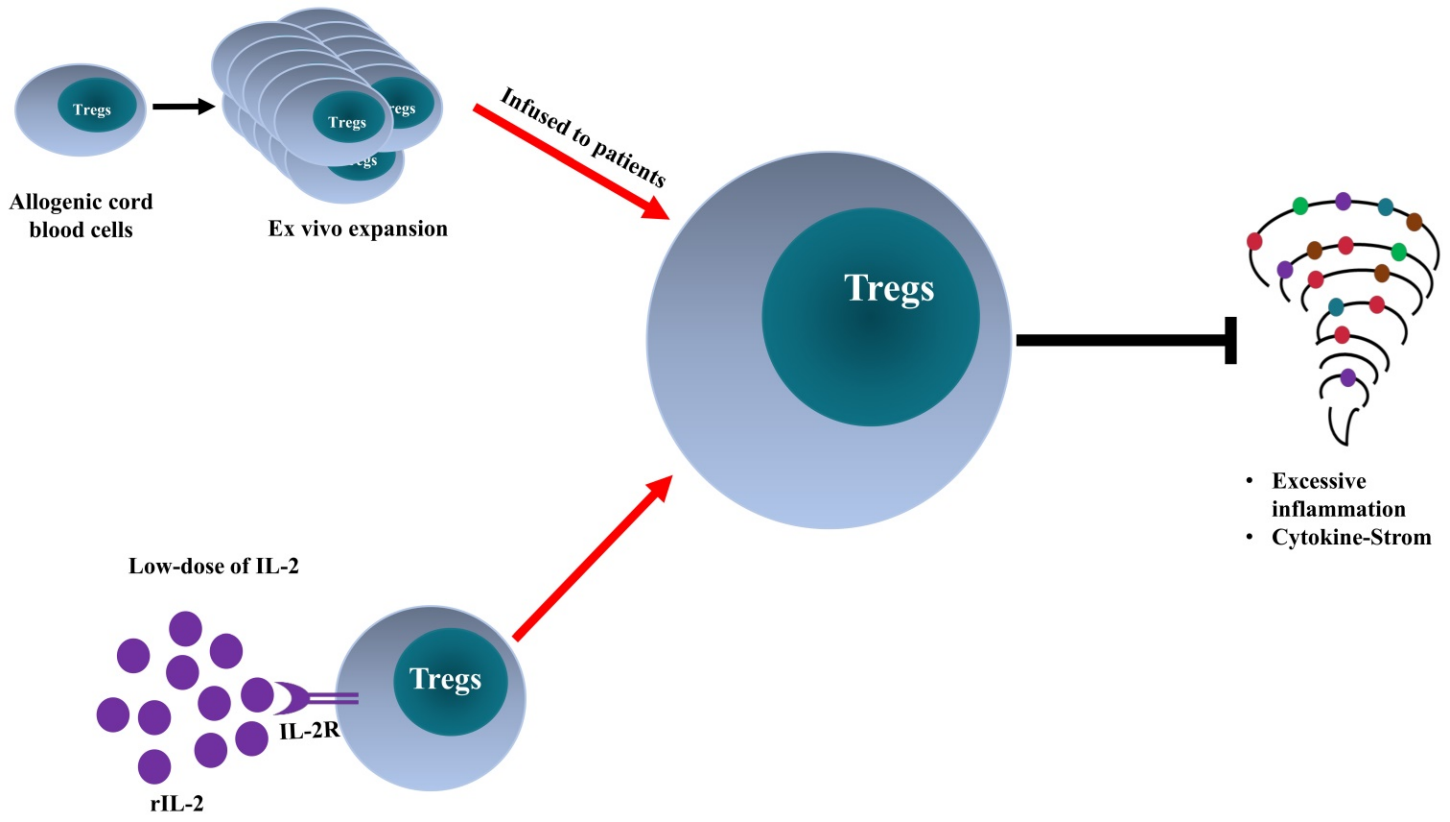
**Comparison of Treg-targeted treatments for COVID-19.** Adoptive transfer of *ex vivo* activated and expanded allogeneic cord-blood derived Tregs and low-dose of recombinant interleukin-2 (rIL-2) enhance Treg activity in severe COVID-19 patients, consequently, dampen the excessive inflammation and quench cytokine storm.

The clinical manifestations of patients infected with SARS-CoV-2 are caused by the cytopathic effects of the virus and by excessive inflammatory responses. Treg cells may be involved in the attenuation of antiviral defense at the early stage of infection and inhibition of cytokine storm in critically ill patients. The exact role of Tregs in the pathogenesis of COVID-19 should be further defined. Treg-based therapy appears to be an innovative and promising approach (schematically shown in Figure 2). Nevertheless, its efficacy and safety should be carefully investigated. Furthermore, other therapeutic agents used in COVID-19 treatment may be attributable to their Treg-boosting effect, and this possibility merits future investigation.

## Figures and Tables

**Table 1:** Summary of current studies on Treg cells in COVID-19 patients

Number of patients or healthy donor controls	Source of Tregs	Markers of Tregs	Changes of Tregs	References
N=57 (7 mild, 26 severe and 24 recovered cases)	PBMCs	CD25^+^FoxP3^+^	Increase (proportion) in severe cases	66
N=4 (mechanically ventilated cases)	BALF and PBMC	FoxP3^+^	Increase (proportion, both in lungs and blood)	53
N=39	PBMC	CD4^+^CD25^+^CD127^lo/-^	Increase (proportion)	160
N=169 (80 mild, 22 severe, 61 mild-recovery and 6 severe-recovery cases)	PBMC	CD4^+^CD25^+^CD127^lo^	Increase (number and proportion)	50
N=1/11 (1 asymptomatic case and 11 healthy controls)	PBMC	CD4^+^CD25^+^CD127^-^ CD45RA^-^FoxP3^hi^	Increase (proportion)	57
N=12/12 (4 mild, 5 severe, 3 critical cases and 12 heathy controls )	PBMC	CD4^+^CD25^+^CD127^-^	Increase (proportion, in mild and severe cases) Decrease (proportion, in critical cases)	51
N=40 (22 Severe, 18 milder disease and 9 ICU cases)	UMAP	FoxP3	Decrease (proportion)	61
N=30/8 (30 cases and 8 healthy controls)	PBMC	CD4^+^FoxP3^+^CD25^+^	Decrease (mRNA level)	60
N=452 (166 mild or moderate and 286 severe cases)	PBMC	CD3^+^CD4^+^CD25^+^ CD127^lo^	Decrease (numbers)	11
N=40 (ICU cases)	PBMC	CD4^+^FoxP3^+^CD25^+^	Decrease (proportion and mRNA level)	63
N=19 (Pericardial effusion cases)	PBMC	CD3^+^CD4^+^CD25^+^CD127^-^	Decrease (proportion)	64
N=19/18 (19 Children cases and 18 healthy controls)	PBMC	CD3^+^CD4^+^CD25^+^CD127^-^	Decrease (proportion, in acute phase) No change (proportion, in convalescent phase)	65
N=109/98 (109 convalescent cases and 98 healthy controls)	PBMC	CD25^+^CD127^-^FoxP3^+^	Decrease (number)	59
N=99 (93 moderate, 1 severe and 5 critical cases)	PBMC	CD4^+^ CD25^+^FoxP3^+^	Decrease (proportion)	62
N=22/10 (11 covid-19 and 11 no-covid-19 cases with cancer, and 10 healthy controls)	PBMC	CD4^+^CD25^+^CD127^-^	No change (proportion)	69
N=52 (17 moderate, 27 severe and 8 critical cases)	PBMC	CD4^+^CD25^+^FoxP3^+^	No change (proportion and number)	67
N=2/2 (A 22-year-old immunocompetent boy, a 63-year-old female COVID cases and 2 healthy controls)	PBMC	CD4^+^CD25^+^CD127^-^ FoxP3^+^	No change (proportion)	68

**Table 2:** Comparison of Treg-targeted treatments

Treatment	Advantages	Disadvantages	References
Adoptive Transfer of Tregs	Infusion possible to allogeneic patients, off-the-shelf “living drug” Large scale expansion *in vitro*; expansion rate is much higher than *in vivo* Phenotypical and functional features can be analyzed prior to infusion Dosage can be precisely controlled	Requires 2-3 weeks for o control for possible phenotypical and functional change after infusion Requires specific facility	161, 162
Low-dose of rIL-2	Effectively expand of Tregs in autoimmune patients Therapeutic agent readily available Applicable in all health care settings	May activate CD25-expressing proinflammatory T cells	163, 164

**Table 3:** COVID-19 therapeutic agents and their Treg-boosting effect

Therapeutic agent	Effect on COVID-19	Action on Tregs	References
Dexamethasone	Improves the outcomes of critically ill patients with ARDS (NCT04445506) Reduces mortality (NCT04381936) Reduces duration for mechanical ventilation (NCT04327401)	Increases in the proportion of Tregs and the ratio of Treg/effector T cells Increases the number of with combination of IL-2 Enhances the function of Tregs in human patients	85, 87, 89-91, 165
Vitamin D	Reduces mortality Reduces the need for ICU treatment Deficiency is associated with increased COVID-19 risk	Induces generation of tolerogenic DCs, IL-10-producing CD4 T cells and antigen-specific Tregs Increases Tregs/T cells ratio and enhances Treg function Increases Foxp3 expression in Tregs and increases Treg number	92, 104, 108, 166-168
Tocilizumab and Sarilumab	Reduces the incidence or duration of ICU and reduces length of hospital stay Rapidly inhibits excessive inflammation (NCT04331795)	Increases the proportions of CD4^+^CD25^+^CD127^lo^ Tregs and HLA-DR^+^ activated Tregs Tips the balance of Th17/Tregs toward Tregs cells	118, 169-171
Chloroquine and hydroxychloroquine	Possesses antiviral activity, inhibits SARS-CoV-2 infection (*in vitro*) Reduces hospitalization rate among mild outpatients	Promotes the expansion and increases the number of Treg cells Promotes Foxp3 expression and differentiation of Treg cells Rebalances Th17/Treg cells	125, 126, 172-174
Azithromycin	Further reduces viral load used together with hydroxychloroquine	Promotes Treg phenotype including Foxp3 expression in bulk Tregs	123, 127, 175
Tetrandrine	Inhibits the entry of SARS-CoV-2 spike-protein pseudovirions (*in vitro*)	Increases the expression of mTNF on APC and induces expansion of Tregs through TNFR2 Inhibit differentiation of Th1, Th2 and Th17 cells, but sparing Treg differentiation	139, 144, 145
Adalimumab	Inhibits the progression of severe COVID-19	Increases mTNF expression on monocyte and promotes expansion of Tregs through TNFR2	132, 134, 137

## Abbreviations

APC: Antigens presenting cells
ARDS: Acute respiratory distress syndrome
COVID-19: Coronavirus disease 2019
EAE: Experimental autoimmune encephalomyelitis
mTNF: Transmembrane TNF
PD-1: Programmed cell death protein 1
SARS-CoV-2: Severe acute respiratory syndrome coronavirus 2
Th1: T helper 1 cells
Th2: T helper 2 cells
Th17: T helper 17 cells
TNF: Tumor necrosis factor
TNFR2: TNF receptor type II
Treg: CD4^+^Foxp3^+^ regulatory T cell
